# Circulating tumor DNA in hepatocellular carcinoma: trends and challenges

**DOI:** 10.1186/s13578-016-0100-z

**Published:** 2016-05-11

**Authors:** Jia-Cheng Tang, Yi-Li Feng, Tao Guo, An-Yong Xie, Xiu-Jun Cai

**Affiliations:** Zhejiang Province Key Laboratory of Laparoscopic Technology, Sir Run Run Shaw Hospital, Zhejiang University, Hangzhou, People’s Republic of China; Institute of Translational Medicine, Zhejiang University, Hangzhou, People’s Republic of China

**Keywords:** Cell free DNA, Circulating tumor DNA, Biomarker, Liquid biopsy, Hepatocellular carcinoma

## Abstract

Molecular characterization of individual patients’ tumor cells is becoming increasingly important in offering effective treatment for patients in clinical practice. Recent advances in the field have indicated that circulating tumor DNA (ctDNA) has huge potential to serve as a biomarker for early detection and precision treatment as well as prognosis of hepatocellular carcinoma (HCC). As ctDNA in HCC patients harbors the molecular characteristics of HCC tumor cells, ctDNA analysis in the blood may be sufficient for convenient, non-invasive and accurate detection, providing information for HCC diagnosis, treatment and prognosis. In this review, we will summarize and discuss current trends and challenges of ctDNA application in HCC.

## Background

Liver cancer, with 782,500 new cases and 745,500 deaths occurring worldwide in 2012, is the second leading cause of global cancer death, with China alone accounting for about 50 % of the total number of cases and deaths [1]. It was estimated that 466,100 new cases and 422,100 deaths would occur in China in 2015, accounting for about 15 % of all cancer deaths in China [2]. Primary liver cancer includes three histologic subtypes, hepatocellular carcinoma (HCC), cholangiocarcinoma and combined hepatocellular and cholangiocarcinoma. HCC, which is the major histological subtype of primary liver cancers, accounts for between 85 and 90 % of all cases worldwide [3]. A key to effective prevention and treatment of HCC is early diagnosis of HCC.

The diagnosis of HCC is increasingly made with the use of noninvasive imaging tests such as ultrasonography, computed tomography (CT) and magnetic resonance tomography (MRI), along with use of an alpha-fetoprotein (AFP) level, a predictive biomarker for HCC. Imaging tests can only determine HCC with confidence to some degree when nodules, benign or malignant, grow to at least 1 cm in size. Invasive biopsy is considered for diagnosis of HCC when imaging tests are less assuring. In either case, patients may by then have malignant tumors in an advanced stage, with limited treatment options and poor prognosis. Meanwhile, it is recognized that not all HCC can produce a higher level of AFP [4]. In fact, if early-stage HCC, currently difficult to diagnose and characterize, can be detected, it can be effectively treated by surgical resection with an 5-year survival rate of 90 % [3]. In addition to surgical resection, several options, including liver transplantation, transarterial chemoembolization (TACE), radiofrequency ablation, high-intensity focused ultrasound and targeted molecular therapy (e.g. sorafenib treatment), are currently used in the clinic to treat HCC. The effectiveness of these treatments can be significantly improved with early detection and convenient monitoring for possible HCC relapse following treatment. Thus clinician and scientists in the field have been actively developing sensitive, reliable and convenient methods for surveillance and early detection of HCC and post-treatment monitoring of HCC relapse.

Hepatocellular tumorigenesis is a slow, progressive and complex process due to the accumulation of genetic and epigenetic alterations in hepatocytes whose activities intimately interact with surrounding microenvironment [5]. Despite serious challenges in detecting these genetic and epigenetic alterations, one method that holds great promise is the detection of circulating tumor DNA (ctDNA) in the peripheral blood of HCC patients. This method, along with circulating tumor cells, is termed “liquid biopsy”. By decoding the information of nucleic acids from patients’ serum or plasma, not only can clinicians make accurate diagnosis and proper treatment on HCC patients, but the scientists can also use this “liquid biopsy” technology to better understand the biology of HCC and help clinicians to design cancer therapy.

## ctDNA of HCC

**Fig. 1:**
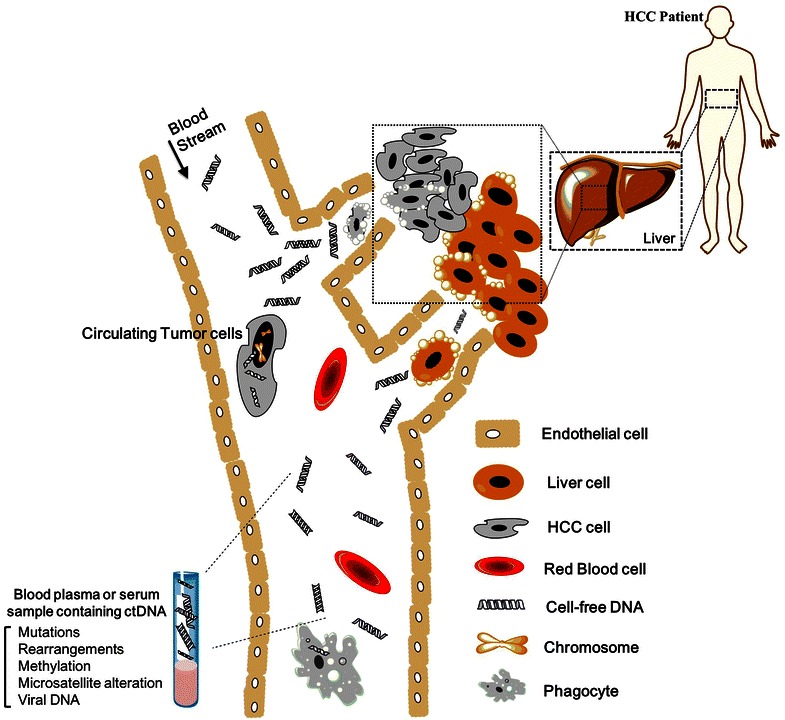
ctDNA release and extraction in HCC patients. ctDNA is released from HCC cells undergoing apoptosis or necrosis and can be extracted from a blood sample. Genetic and epigenetic aberrations in ctDNA can be detected and quantified. These genetic alterations include mutations, rearrangements, methylation, microsatellite alteration and integrated viral DNA. The detection of these alterations in the background of “normal” cfDNA molecules in principle offers a higher diagnostic specificity in comparison with only quantitative measurement of total cfDNA alone

Among varieties of circulating cell-free DNA (cfDNA), ctDNA is released into circulation specifically from tumor cells that undergo metabolic secretion, apoptosis or necrosis (Fig. 1). cfDNA is defined as extracellular DNA present in plasma or serum samples. It can be detected not only in patients suffering from cancer or other diseases but also in healthy individuals. Unlike normal cfDNA, ctDNA carries tumor-specific genetic or epigenetic alterations, such as point mutations, copy number variations, chromosomal rearrangements, DNA methylation patterns, etc. Thus, a minimally invasive examination of ctDNA with a small amount of peripheral blood from patients could reveal genetic and epigenetic alterations related to specific cancer and its metastatic state, offering a unique opportunity for serially monitoring tumor genomes in a non-invasive, convenient and accurate manner.

**Fig. 2:**
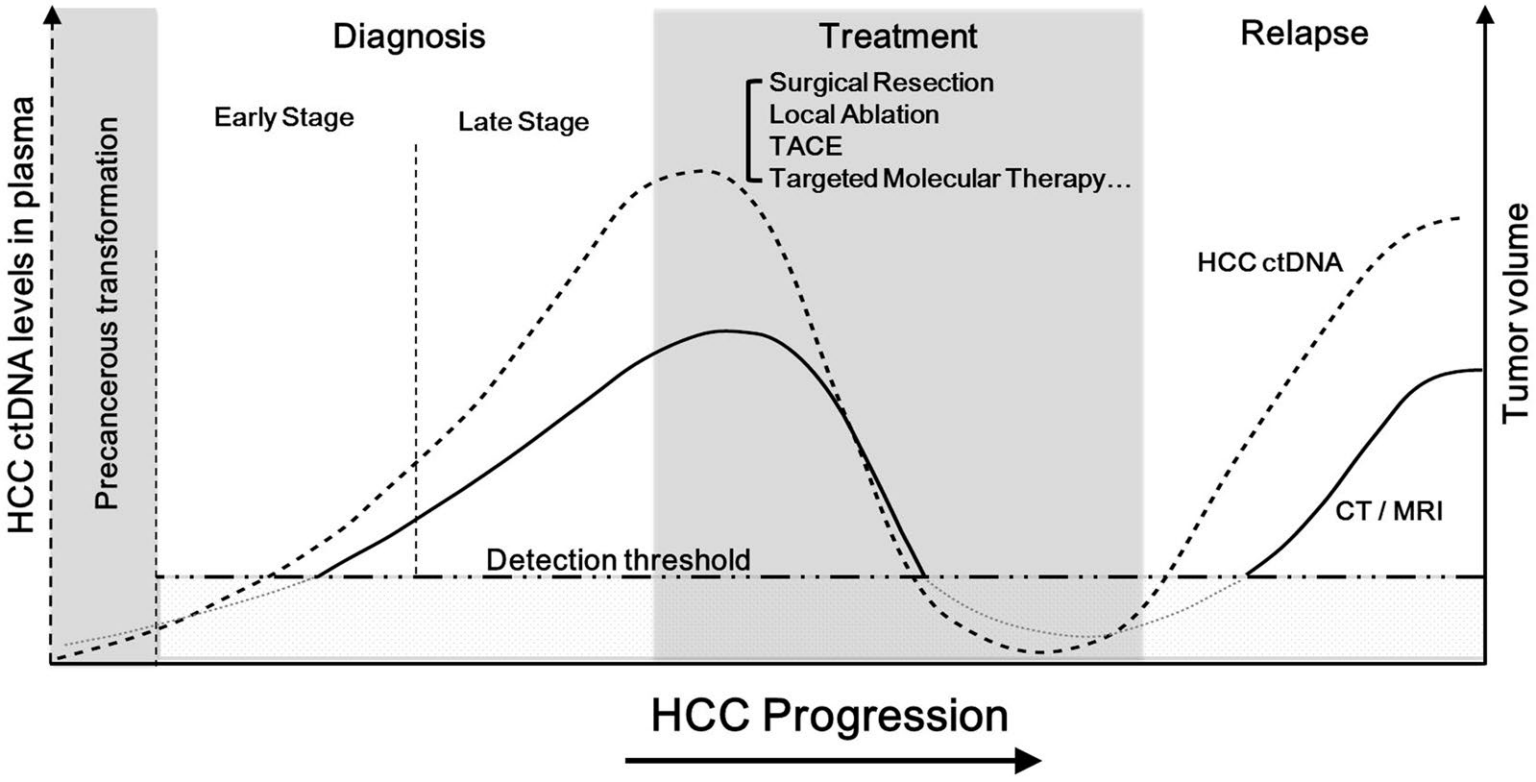
Monitoring response and relapse of HCC patients with targeted therapies by ctDNA detection. From early stage lesion to late phase of carcinogenesis, an excess of apoptotic cell death, as occurs in large and rapidly proliferating HCC tumors (*solid line*), can lead to an increase of ctDNA levels in plasma (*dash line*). Moreover, the levels of ctDNA correlate well with HCC progression as well as various therapy including surgical resection, local ablation, TACE and targeted molecular therapy

Since 2006, cancer genome sequencing has delivered robust and comprehensive tumor genome information, which provides a fertile ground for the development of ctDNA testing. Along with the advancement of sequencing technology, huge effort and investment are being taken worldwide in putting this “liquid biopsy” into clinical practice. Potential applications of ctDNA testing in HCC patients may include: (a) early detection of cancer; (b) monitoring of tumor heterogeneity and metastasis; (c) identification of therapeutic targets; (d) real-time evaluation of treatment response and tumor relapse; and (e) real-time assessment of evolution of drug resistance (Fig. 2). These applications require detection of genetic and epigenetic alterations in ctDNA specifically associated with different stages of HCC (e.g. hepatocellular dysplasia, early HCC, progressed HCC and metastatic HCC) and with different treatment options or stages of treatment. However, progress is limited in exploring the clinical utility of ctDNA in cancer diagnosis, treatment and prognosis, particularly in HCC, due to serious technological obstacles in detection and analysis of ctDNA.

## Detection and analysis of ctDNA in HCC patients

The pioneer work by Mandel and Metais welcomed the discovery of cfDNA in 1948 [6]. But during the following two decades, we witnessed the futility in this field. Until 1977, by comparing the blood samples between 173 cancer patients and 53 healthy individuals using a radioimmunoassay that can detect DNA in nanogram, Leon et al. found the cancer patients had a relative higher level of cfDNA than healthy controls and an increased level after radiation therapy [7]. This finding exhibited the potential of cfDNA as a cancer biomarker, which can also be applied to patients with HCC. In 1989, ctDNA was noted to be a fraction of cfDNA in the blood [8]. Two groups recently found that the cfDNA levels in patients with hepatitis C virus (HCV)-related HCC were correlated with the overall survival and extrahepatic recurrence in distant organs after curative hepatectomy [9, 10]. In addition, several studies have examined global alterations of cfDNA, such as DNA methylation alterations, microsatellite alterations, point mutations, chromosomal rearrangements and viral DNA integration (Table 1), some of which have genetic and epigenetic alteration patterns similar to those detected in primary tumors. Among a pool of cfDNA, ctDNA likely harbors these alterations, demonstrating the potential utility of ctDNA in clinical applications.

**Table 1:** Detection of cfDNA and its alterations in HCC patients

Forms	Gene	Tumor	cfDNA	Diagnostic/prognostic		Notes
Methylation	RASSF1A [17] (combined AFP)	59/63 (93 %)	12/22 (60 %)	*√*	*√*	Associated with HCC
	GSTP1 [15]	23/26 (88.5 %)	14/23 (61 %)	*√*	*√*	Associated with HCC
	*p15* promoter [13]	16/25 (64 %)	4/16 (25 %)	*√*	*√*	*p15/p16* methylation in the plasma/serum was highly associated with HCC
	*p16* [16]	16/22 (73 %)	13/16 (81 %)	*√*	*√*	
Microsatellite alterations	D8S258 and D8S264 [25]	Sensitivity (51.9 %) Specificity (77.5 %)		NA	*√*	Associated with HCC metastasis
Mutation	Ser-249 p53 [34]	NA	74/186 (39.8 %)	*√*	*√*	Aflatoxin-associated mutation and chronic infection with HBV, multiplicative associated with HCC
DNA integrity	LINE-1 hypomethylation [19]	NA	NA	*√*	*√*	Associated with HCC
Viral DNA	HBV DNA [39]	NA	NA	*√*	NA	Associated with TAE and lipiodol retention
/	Inflammatory cytokine genes [9]	NA	NA	NA	*√*	Amount higher in HCV-related HCCs than in HCV carriers

* Includes all articles published to date that assess cfDNA detection and alterations of cfDNA in HCC patients, which were summarized and clarified the significance

### Methylation alterations

Methylation alterations occur on many genes associated with initiation and progression of HCC. Several studies have revealed the alterations of DNA methylation in HCC patients’ tumor tissues including the aberrant methylation of the promoter region of Glutathione S-transferase P1 (*GSTP1*) [11, 12] and the cyclin-dependent kinase inhibitor *p15* [13] and *p16* [14]. Efforts have been made to detect such methylation alterations of cfDNA in HCC patients’ blood. The successful detection of hypermethylated *GSTP1* [15], *p15* [13] and *p16* [16] in cfDNA from HCC patients may allow the development of a blood-based assay for HCC diagnosis. Methylation alterations of RAS association domain family 1A (*RASSF1A*) were also detected in cfDNA of HCC patients and account for 40 % cases of matched plasma [17]. Furthermore, hepatitis B virus (HBV) carriers undergoing surveillance and subsequently developing HCC had significantly higher levels of *RASSF1A* from the time of enrollment to cancer diagnosis [18]. Another gene with methylation abnormality that has been detected in HCC patients is long interspersed nucleotide elements (*LINE*-*1*) [19]. The progression and invasiveness of HCC are highly associated with elevated hypomethylated *LINE*-*1*. These lines of evidence indicate that a combined assessment of circulating methylated DNA may yield a promising tool in HCC diagnosis and management.

Methylation-based assays on ctDNA may offer the best hope for early detection, as some of methylation changes are mechanistically early events in tumor progression. Unlike genetic alterations such as mutations and deletions, epigenetic changes are also potentially reversible [20] and can therefore be targeted for prevention of tumor initiation and progression. Compared with the detection of cfDNA with microsatellite instability as described below, the methylation-based approach is more sensitive and generates fewer false negatives [16]. In contrast, mutations occur in a large number and in varied frequencies, and many of highly frequent mutations are associated with different cancers. This complicates the use of mutations for HCC diagnosis by ctDNA analysis. It appears that the methylation approach is better suited at present for HCC screening using plasma or serum samples.

### Microsatellite alterations

Highly polymorphic DNA repeat regions, termed microsatellites, are commonly present in eukaryotic genomes. Loss and length alteration in microsatellites are frequent in a variety of cancers, providing a set of markers suitable for diagnostic detection. Since the discovery of tumor-derived microsatellite alterations in cfDNA [21, 22], interest in uncovering these alterations of cfDNA is growing. The comparative genomic hybridization (CGH) technique has enabled scientists to define some microsatellite alterations in HCC such as chromosome 8p, 17p and 19p deletions [23, 24], and the loss on 8p and 19p might contribute to HCC metastasis [23, 25].

In the early 20th century, a microsatellite marker screen was performed in primary tumor and serum sample in 21 cases of HCC patients, 76.2 percent of which harbor at least one allelic imbalance [26], providing early evidence for clinical utility of this approach. A combined set of microsatellite loci gives a higher probability of detecting a risk population. Two microsatellite markers on chromosome 8p D8S258 and D8S264 have been determined as contributors to HCC metastasis by comparing primary tumors and matched metastases [23]. Interestingly, only an allelic imbalance at D8S258 was found in the cfDNA of HCC patients, and combination of both the allelic imbalance and a higher level of cfDNA is well correlated with the decrease in disease-free and overall survival rates [24].

### Point mutations

Tumor progression involves the accumulation of both inactivation of tumor suppressor genes and activation of proto-oncogenes, for instance, *TP53* and *CTNNB1* in HCC. Ser249 of *TP53* is the most reported mutation hotspot in HCC patients, and mutation of this site leads to the deficiency in its specific DNA binding ability [27–32]. Recently, *TP53* Ser249 mutant in plasma has been reported to be highly associated with cirrhosis and HCC in China and Africa [33–35], a region with high HBV prevalence and high Aflatoxin B1 exposure. Interestingly, this mutation was also detected in noncancerous hepatic tissues of HCC [36], in plasma DNA of a few healthy individuals and in patients with relatively more severe cirrhosis [34], indicating this mutation might be involved in early development of HCC and accumulate during HCC progression. However, since this mutation, like many other mutations, occurs in other types of cancers, it cannot be excluded that ctDNA harboring this mutation is released from other tissues.

### Chromosomal rearrangements

Genomic sequencing has revealed many recurrent chromosomal rearrangements including deletions, insertions, amplifications, translocations and more complex rearrangements in HCC. Detection of such chromosomal rearrangements in circulating tumor cell requires highly sensitive PCR. To date, rearrangement detection assay in plasma has just succeeded in a small population of patients, mostly in hematological malignancies. Nevertheless, whole genome sequencing of ctDNA provides the opportunity to identify chromosomal rearrangements or copy number changes in HCC patients, and ultimately offer a reliable and robust method for HCC detection. In addition, some non-coding DNA, such as *LINE*-*1* which distributes throughout the genome, significantly increases in the serum of patients with HCC, in a hypomethylated form [19], providing a target for ctDNA detection. Several other DNA abnormalities have also been investigated. For instance, the presence of longer relative telomere length (RTL) predicts an elevated risk for non-cirrhotic HCC patients with HBV [37] while telomere shortening occurs in cirrhosis samples. This difference could be used as a biomarker in blood to distinguish stages of severe liver diseases.

### Viral DNA

Virus infection is the major contribution to severe liver diseases. HBV and HCV are important etiological factors for HCC, and the combined fraction of cases attributable to virus infection is estimated to 75 % of all HCC cases. The specific virus DNA level may potentially be used as molecular biomarkers of disease activity. High level of serum HBV DNA has a strong association with the incidence of HCC. Interestingly, the HBV DNA-based prediction is independent of the level of hepatitis B surface antigen or alanine aminotransferase level, and the presence of cirrhosis [38], making it a sensitive and reliable tool for monitoring the disease. The detection of circulating HBV DNA is also applied for patients who underwent transcatheter arterial embolization (TAE). TAE is an important palliative treatment for HCC patients who are poor candidates for surgery or percutaneous ablative therapy. A recent study showed that the elevated plasma HBV DNA persistently correlates with lipiodol retention, but not with age or tumor size, making it an early indicator to assess the success or failure of TAE [39]. In addition, some conservative mutations in HBV provide new targets in plasma detection, such as a double 1762T/1764A mutation in HBV genome in HCC tumors from Qidong area, China [40].

## Challenges in clinical utility of ctDNA in HCC

It has been demonstrated that the concentration of ctDNA in the plasma increased along with stages of several human malignancies [41], providing potential utility of ctDNA in diagnosing advanced stages of cancer and monitoring cancer relapse following cancer treatment, especially for breast cancer and lung cancer patients [42, 43]. In a recent study, ctDNA-based detection preceded clinical detection of metastasis for 86 % patients with an average lead time of 11 months following primary surgery for primary breast cancer patients [44]. However, it remains a challenge in applying the ctDNA technology in early detection of cancer including HCC. Even for advanced stage of HCC, this “liquid biopsy” methodology is yet to be established for clinical applications largely due to low level of ctDNA, poorly characterized genetic and epigenetic alterations in HCC patients and high level of tumor heterogeneity of HCC.

### Low levels of ctDNA

Over past decades, many methodologies, such as Sanger sequencing (dideoxy-terminator sequencing), pyrosequencing, next-generation sequencing, real-time PCR, and amplification refractory mutation system (ARMS), have been employed to detect genetic alterations in tumor tissues. However, these methodologies are not sensitive or accurate enough to quantitatively assess cfDNA in cancer patients for clinical purposes due to several limitations. First, cfDNA is only in trace amount in the serum and plasma of cancer patients and is too low to be efficiently isolated. Secondly, ctDNA represents only a very small fraction of cfDNA, making it extremely difficult to detect. In addition, the increase in cell death caused by tumor-promoting inflammation and/or tissue repair processes dilutes tumor-specific genetic and epigenetic alterations by adding more small, fragmented “background” DNA, as well as proteins and other molecules, into blood, making it impossible for consistent detection and accurate assessment of ctDNA using the above existing technologies. As a result, the clinical utility of ctDNA in diagnosing and monitoring patients with cancers including HCC has been limited.

In order to reliably enrich, detect and analyze ctDNA, new methodologies, such as PCR-based digital assays [45] and DNA sequencing-based assays, have been developed. PCR-based digital assays include droplet digital PCR [46] and BEAMing on the basis of four components (beads, emulsion, amplification, and magnetics) [47], whereas DNA sequencing-based assays include pyrophosphorolysis-activated polymerization (PAP) [48], tagged-amplicon deep sequencing (TAM-Seq) [49], safe-sequencing system (Safe-SeqS) [50], cancer personalized profiling by deep sequencing (CAPP-Seq) [43] and personalized analysis of rearranged ends (PARE) [51, 52] (Table 2). BEAMing and digital droplet PCR have been developed for diagnosing patients with breast cancer [42], colon cancer [53, 54] and gastric cancer [55]. CAPP-Seq has successfully identified 85 % of non–small-cell lung cancer (NSCLC) patients with stage II–IV and 50 % of patients with stage I NSCLC [43]. A later study compared digital sequencing of plasma-derived cfDNA to tissue-based sequencing on 165 consecutive matched samples in solid tumor cancers, proved the clinical sensitivity was 85.0 and 80.7 %, respectively [56]. These new advances have expanded our ability to detect tumor-specific genetic and epigenetic alterations including DNA methylations, point mutations, amplifications, chromosomal rearrangements, and aneuploidy in ctDNA. Recently, the approach termed shotgun massively parallel sequencing was applied to establish correlation between the fractional concentrations of ctDNA and the tumor size and surgical treatment [57]. Notably, this approach has the ability to scan genome-wide landscape ranging from genomic aberrations to point mutations. Despite these technological advances, the low level of ctDNA remains a major factor that limits the utility of ctDNA in HCC diagnosis. The field urgently needs a method to efficiently enrich ctDNA from a pool of cfDNA.

**Table 2:** Potential methods for HCC ctDNA detection

Technique	Detection capability (mutant DNA/total DNA) (%)
Sanger sequencing	>10
Pyrosequencing	10
Next-generation sequencing	>1
Real-time	1
ARMS	0.1
Digital droplet PCR, BEAMing, PAP, TAM-seq, Safe-SeqS, CAPP-seq, PARE	<0.01

### Poorly characterized DNA alterations of HCC

Cancer somatic alterations form the basis for ctDNA detection and analysis. DNA alterations harbored in ctDNA reflect those occurring in tumors. However, unlike breast cancer or lung cancer, which has multiple well-defined genetic aberrations that dictate tumor behavior, the profiles of genetic and epigenetic alterations in HCC are poorly characterized. To date, qualitative analysis of abnormal concentrations of ctDNA or single-gene methylation alterations alone is not recommended for HCC diagnosis base on a meta-analysis, while combining with AFP improves the diagnostic performance [58]. During the recent 5 years, the comprehensive genome-wide deep sequencing of HCC tumor samples led to identification of many HCC-specific driver mutations, including aberrations in *TERT* promoter, *TP53*, *CTNNB1*, *ARID1A*/*ARID1B*, *Axin1*, *APC* (adenomatous polyposis coli), *TSC2* and many others (Lin and Cai, manuscript in preparation) [59–63]. Mapping somatic changes in HCC tumors, in combination with other HCC-specific chromosomal rearrangements and epigenetic alterations, may pave the way for development of ctDNA detection and analysis technologies for HCC patients.

### HCC heterogeneity

Genetic and epigenetic profiles vary in different populations of tumor cells within the same primary tumor, as well as their metastases from the same patient. This phenomenon was termed “tumor heterogeneity”, which poses serious clinical barriers to targeted therapy. The development of tumor heterogeneity is attributed to clonal evolution associated with acquisition of differential genetic and epigenetic alterations. In HCC, different genetic and epigenetic alterations in individual tumor cells, together with selection pressure upon them, may cause populations of tumor cells within a tumor to undergo molecularly heterogeneous transformation, even with the seemingly identical histopathological traits [64, 65]. This evolution can start at varying times points during initiation and progression of a tumor and/or at varying sites within a tumor, resulting in the spatial and temporal heterogeneity of HCC. As a result, a single-site biopsy is certain to miss clinically important mutations from a heterogeneous HCC tumor [66, 67]. In contrast, ctDNA analyzed by a personalized ctDNA detection approach is a pool of DNA fragments released from nearly every part of a tumor and every tumor in the patient and maintains tumor heterogeneity in term of genetic and epigenetic alterations. The capability of dissecting tumor heterogeneity, along with convenience and less invasiveness, makes the ctDNA approach much more desirable in diagnosis of cancers, including HCC. A proof-of-concept study has been taken recently that analysis of ctDNA can monitor somatic genetic alterations during the tumor progression, covering the whole geographical region of the tumor. In this study performed on a 66-year-old woman, 16 mutation signatures in liver metastasis and nine in primary tumor were identified, all of which were detected in ctDNA [68]. Although carried out in one single patient, this attempt presents a promising approach for overcoming the clinical challenges of heterogeneity and improving therapeutic effectiveness.

## Conclusions

Although several protein-based HCC biomarkers have been reported, very few of them demonstrate solid diagnostic performance [69]. In future medical management, more patients will allow their physicians to make therapeutic decisions guided by genetic analysis of ctDNA. A considerable amount of studies make it clear that clinicians are entering the age in which ctDNA analysis will be a key part of tumor management, as this convenient, non-invasive and accurate diagnostic approach will reduce the anxiety of patients, as well as clinicians, and help prevent cancer progression or even cure cancer.

But prior to the clinical applications, a mechanistic understanding of the biology of ctDNA is urgently needed. Previous efforts revealed that HCC patients mostly contain genomic DNA in plasma [70], but recent work uncovered that the tumor-associated aberrations preferentially distribute in short DNA molecules [71]. Such evidence helps improve our understanding of the nature of ctDNA and might provide guideline for technology improvement and clinical practice. In addition, it is unclear to what extent the spectra of genetic and epigenetic alterations of ctDNA resemble those in tumors of a patient. Our understanding in this direction will help determine the scope of future applications of ctDNA.

Technological advances in ctDNA enrichment and analysis help expand the potential of the ctDNA-based liquid biopsy in cancer diagnosis and therapy. However, current technologies for ctDNA enrichment and analysis are yet to work in early detection of cancer due to fast decay of ctDNA and extremely low amount of ctDNA from early stage of HCC. Identification of the increasing number of alterations in ctDNA of early HCC makes it possible to improve ctDNA enrichments and analysis for early detection of HCC, which will benefit patients the most when the tumor will be most amenable to cure. Further, due to needle biopsy sampling bias and the limited availability of “research biopsies” in advanced cancer patients, the investigation of HCC metastasis and drug resistance has been challenging. It is believed that ctDNA carries different genetic or epigenetic signatures when tumor cells in a HCC patient become metastatic or when a patient develops drug resistance. It is therefore desirable to take repetitive monitoring of these signatures (or events) in ctDNA using blood samples and have sufficient information to devise treatment options. Together, our efforts in these cDNA-related areas would provide many healthcare advances and improve the life of cancer patients.

## Abbreviations

CAPP-Seq: cancer personalized profiling by deep sequencing
cfDNA: cell-free DNA
CT: computed tomography
ctDNA: circulating tumor DNA
GSTP1: glutathione S-transferase P1
HBV: hepatitis B virus
HCV: hepatitis C virus
HCC: hepatocellular carcinoma
LINE-1: long interspersed nucleotide elements
MRI: magnetic resonance tomography
RASSF1A: RAS association domain family 1A
TACE: transhepatic arterial chemotherapy and embolization
TAE: transcatheter arterial embolization

## References

[1] Torre LA, Bray F, Siegel RL, Ferlay J, Lortet-Tieulent J, Jemal A (2015). Global cancer statistics, 2012. CA Cancer J Clin.

[2] Chen W, Zheng R, Baade PD, Zhang S, Zeng H, Bray F, Jemal A, Yu XQ, He J (2016). Cancer statistics in China, 2015. CA Cancer J Clin.

[3] El-Serag HB, Rudolph KL (2007). Hepatocellular carcinoma: epidemiology and molecular carcinogenesis. Gastroenterology.

[4] Attwa MH, El-Etreby SA (2015). Guide for diagnosis and treatment of hepatocellular carcinoma. World J Hepatol.

[5] Kanda M, Sugimoto H, Kodera Y (2015). Genetic and epigenetic aspects of initiation and progression of hepatocellular carcinoma. World J Gastroenterol.

[6] Mandel P, Metais P (1948). Les acides nucléiques du plasma sanguin chez l’homme. C R Seances Soc Biol Fil.

[7] Leon SA, Shapiro B, Sklaroff DM, Yaros MJ (1977). Free DNA in the serum of cancer patients and the effect of therapy. Cancer Res.

[8] Stroun M, Anker P, Maurice P, Lyautey J, Lederrey C, Beljanski M (1989). Neoplastic characteristics of the DNA found in the plasma of cancer patients. Oncology.

[9] Iida M, Iizuka N, Sakaida I, Moribe T, Fujita N, Miura T, Tamatsukuri S, Ishitsuka H, Uchida K, Terai S (2008). Relation between serum levels of cell-free DNA and inflammation status in hepatitis C virus-related hepatocellular carcinoma. Oncol Rep.

[10] Tokuhisa Y, Iizuka N, Sakaida I, Moribe T, Fujita N, Miura T, Tamatsukuri S, Ishitsuka H, Uchida K, Terai S (2007). Circulating cell-free DNA as a predictive marker for distant metastasis of hepatitis C virus-related hepatocellular carcinoma. Br J Cancer.

[11] Tchou JC, Lin X, Freije D, Isaacs WB, Brooks JD, Rashid A, De Marzo AM, Kanai Y, Hirohashi S, Nelson WG (2000). GSTP1 CpG island DNA hypermethylation in hepatocellular carcinomas. Int J Oncol.

[12] Zhong S, Tang MW, Yeo W, Liu C, Lo YM, Johnson PJ (2002). Silencing of GSTP1 gene by CpG island DNA hypermethylation in HBV-associated hepatocellular carcinomas. Clin Cancer Res.

[13] Wong IH, Lo YM, Yeo W, Lau WY, Johnson PJ (2000). Frequent p15 promoter methylation in tumor and peripheral blood from hepatocellular carcinoma patients. Clin Cancer Res.

[14] Matsuda Y, Ichida T, Matsuzawa J, Sugimura K, Asakura H (1999). p16(INK4) is inactivated by extensive CpG methylation in human hepatocellular carcinoma. Gastroenterology.

[15] Wang J, Qin Y, Li B, Sun Z, Yang B (2006). Detection of aberrant promoter methylation of GSTP1 in the tumor and serum of Chinese human primary hepatocellular carcinoma patients. Clin Biochem.

[16] Wong IH, Lo YM, Zhang J, Liew CT, Ng MH, Wong N, Lai PB, Lau WY, Hjelm NM, Johnson PJ (1999). Detection of aberrant p16 methylation in the plasma and serum of liver cancer patients. Cancer Res.

[17] Yeo W, Wong N, Wong WL, Lai PB, Zhong S, Johnson PJ (2005). High frequency of promoter hypermethylation of RASSF1A in tumor and plasma of patients with hepatocellular carcinoma. Liver Int.

[18] Chan KC, Lai PB, Mok TS, Chan HL, Ding C, Yeung SW, Lo YM (2008). Quantitative analysis of circulating methylated DNA as a biomarker for hepatocellular carcinoma. Clin Chem.

[19] Tangkijvanich P, Hourpai N, Rattanatanyong P, Wisedopas N, Mahachai V, Mutirangura A (2007). Serum LINE-1 hypomethylation as a potential prognostic marker for hepatocellular carcinoma. Clin Chim Acta.

[20] Kristensen LS, Hansen LL (2009). PCR-based methods for detecting single-locus DNA methylation biomarkers in cancer diagnostics, prognostics, and response to treatment. Clin Chem.

[21] Nawroz H, Koch W, Anker P, Stroun M, Sidransky D (1996). Microsatellite alterations in serum DNA of head and neck cancer patients. Nat Med.

[22] Chen XQ, Stroun M, Magnenat JL, Nicod LP, Kurt AM, Lyautey J, Lederrey C, Anker P (1996). Microsatellite alterations in plasma DNA of small cell lung cancer patients. Nat Med.

[23] Zhang LH, Qin LX, Ma ZC, Ye SL, Liu YK, Ye QH, Wu X, Huang W, Tang ZY (2003). Allelic imbalance regions on chromosomes 8p, 17p and 19p related to metastasis of hepatocellular carcinoma: comparison between matched primary and metastatic lesions in 22 patients by genome-wide microsatellite analysis. J Cancer Res Clin Oncol.

[24] Ren N, Qin LX, Tu H, Liu YK, Zhang BH, Tang ZY (2006). The prognostic value of circulating plasma DNA level and its allelic imbalance on chromosome 8p in patients with hepatocellular carcinoma. J Cancer Res Clin Oncol.

[25] Qin LX, Tang ZY, Sham JS, Ma ZC, Ye SL, Zhou XD, Wu ZQ, Trent JM, Guan XY (1999). The association of chromosome 8p deletion and tumor metastasis in human hepatocellular carcinoma. Cancer Res.

[26] Chang YC, Ho CL, Chen HH, Chang TT, Lai WW, Dai YC, Lee WY, Chow NH (2002). Molecular diagnosis of primary liver cancer by microsatellite DNA analysis in the serum. Br J Cancer.

[27] Bressac B, Kew M, Wands J, Ozturk M (1991). Selective G to T mutations of p53 gene in hepatocellular carcinoma from southern Africa. Nature.

[28] Hollstein M, Sidransky D, Vogelstein B, Harris CC (1991). p53 mutations in human cancers. Science.

[29] Montesano R, Hainaut P, Wild CP (1997). Hepatocellular carcinoma: from gene to public health. J Natl Cancer Inst.

[30] Jackson PE, Qian GS, Friesen MD, Zhu YR, Lu P, Wang JB, Wu Y, Kensler TW, Vogelstein B, Groopman JD (2001). Specific p53 mutations detected in plasma and tumors of hepatocellular carcinoma patients by electrospray ionization mass spectrometry. Cancer Res.

[31] Kirk GD, Camus-Randon AM, Mendy M, Goedert JJ, Merle P, Trepo C, Brechot C, Hainaut P, Montesano R (2000). Ser-249 p53 mutations in plasma DNA of patients with hepatocellular carcinoma from The Gambia. J Natl Cancer Inst.

[32] Szymanska K, Lesi OA, Kirk GD, Sam O, Taniere P, Scoazec JY, Mendy M, Friesen MD, Whittle H, Montesano R, Hainaut P (2004). Ser-249TP53 mutation in tumour and plasma DNA of hepatocellular carcinoma patients from a high incidence area in the Gambia, West Africa. Int J Cancer.

[33] Kirk GD, Lesi OA, Mendy M, Akano AO, Sam O, Goedert JJ, Hainaut P, Hall AJ, Whittle H, Montesano R (2004). The Gambia liver cancer study: infection with hepatitis B and C and the risk of hepatocellular carcinoma in West Africa. Hepatology.

[34] Kirk GD, Lesi OA, Mendy M, Szymanska K, Whittle H, Goedert JJ, Hainaut P, Montesano R (2005). 249(ser) TP53 mutation in plasma DNA, hepatitis B viral infection, and risk of hepatocellular carcinoma. Oncogene.

[35] Hosny G, Farahat N, Tayel H, Hainaut P (2008). Ser-249 TP53 and CTNNB1 mutations in circulating free DNA of Egyptian patients with hepatocellular carcinoma versus chronic liver diseases. Cancer Lett.

[36] Aguilar F, Harris CC, Sun T, Hollstein M, Cerutti P (1994). Geographic variation of p53 mutational profile in nonmalignant human liver. Science.

[37] Fu X, Wan S, Hann HW, Myers RE, Hann RS, Au J, Chen B, Xing J, Yang H (2012). Relative telomere length: a novel non-invasive biomarker for the risk of non-cirrhotic hepatocellular carcinoma in patients with chronic hepatitis B infection. Eur J Cancer.

[38] Chen CJ, Yang HI, Su J, Jen CL, You SL, Lu SN, Huang GT, Iloeje UH (2006). Risk of hepatocellular carcinoma across a biological gradient of serum hepatitis B virus DNA level. JAMA.

[39] Su YW, Huang YW, Chen SH, Tzen CY (2005). Quantitative analysis of plasma HBV DNA for early evaluation of the response to transcatheter arterial embolization for HBV-related hepatocellular carcinoma. World J Gastroenterol.

[40] Kuang SY, Jackson PE, Wang JB, Lu PX, Munoz A, Qian GS, Kensler TW, Groopman JD (2004). Specific mutations of hepatitis B virus in plasma predict liver cancer development. Proc Natl Acad Sci USA.

[41] Bettegowda C, Sausen M, Leary RJ, Kinde I, Wang Y, Agrawal N, Bartlett BR, Wang H, Luber B, Alani RM (2014). Detection of circulating tumor DNA in early- and late-stage human malignancies. Sci Transl Med.

[42] Dawson SJ, Tsui DW, Murtaza M, Biggs H, Rueda OM, Chin SF, Dunning MJ, Gale D, Forshew T, Mahler-Araujo B (2013). Analysis of circulating tumor DNA to monitor metastatic breast cancer. N Engl J Med.

[43] Newman AM, Bratman SV, To J, Wynne JF, Eclov NC, Modlin LA, Liu CL, Neal JW, Wakelee HA, Merritt RE (2014). An ultrasensitive method for quantitating circulating tumor DNA with broad patient coverage. Nat Med.

[44] Olsson E, Winter C, George A, Chen Y, Howlin J, Tang MH, Dahlgren M, Schulz R, Grabau D, van Westen D (2015). Serial monitoring of circulating tumor DNA in patients with primary breast cancer for detection of occult metastatic disease. EMBO Mol Med.

[45] Vogelstein B, Kinzler KW (1999). Digital PCR. Proc Natl Acad Sci USA.

[46] Hudecova I (2015). Digital PCR analysis of circulating nucleic acids. Clin Biochem.

[47] Diehl F, Li M, He Y, Kinzler KW, Vogelstein B, Dressman D (2006). BEAMing: single-molecule PCR on microparticles in water-in-oil emulsions. Nat Methods.

[48] Madic J, Piperno-Neumann S, Servois V, Rampanou A, Milder M, Trouiller B, Gentien D, Saada S, Assayag F, Thuleau A (2012). Pyrophosphorolysis-activated polymerization detects circulating tumor DNA in metastatic uveal melanoma. Clin Cancer Res.

[49] Forshew T, Murtaza M, Parkinson C, Gale D, Tsui DW, Kaper F, Dawson SJ, Piskorz AM, Jimenez-Linan M, Bentley D (2012). Noninvasive identification and monitoring of cancer mutations by targeted deep sequencing of plasma DNA. Sci Transl Med.

[50] Kinde I, Wu J, Papadopoulos N, Kinzler KW, Vogelstein B (2011). Detection and quantification of rare mutations with massively parallel sequencing. Proc Natl Acad Sci USA.

[51] Leary RJ, Kinde I, Diehl F, Schmidt K, Clouser C, Duncan C, Antipova A, Lee C, McKernan K, De La Vega FM (2010). Development of personalized tumor biomarkers using massively parallel sequencing. Sci Transl Med.

[52] Leary RJ, Sausen M, Kinde I, Papadopoulos N, Carpten JD, Craig D, O’Shaughnessy J, Kinzler KW, Parmigiani G, Vogelstein B (2012). Detection of chromosomal alterations in the circulation of cancer patients with whole-genome sequencing. Sci Transl Med.

[53] Diehl F, Schmidt K, Choti MA, Romans K, Goodman S, Li M, Thornton K, Agrawal N, Sokoll L, Szabo SA (2008). Circulating mutant DNA to assess tumor dynamics. Nat Med.

[54] Taly V, Pekin D, Benhaim L, Kotsopoulos SK, Le Corre D, Li X, Atochin I, Link DR, Griffiths AD, Pallier K (2013). Multiplex picodroplet digital PCR to detect KRAS mutations in circulating DNA from the plasma of colorectal cancer patients. Clin Chem.

[55] Hamakawa T, Kukita Y, Kurokawa Y, Miyazaki Y, Takahashi T, Yamasaki M, Miyata H, Nakajima K, Taniguchi K, Takiguchi S (2015). Monitoring gastric cancer progression with circulating tumour DNA. Br J Cancer.

[56] Lanman RB, Mortimer SA, Zill OA, Sebisanovic D, Lopez R, Blau S, Collisson EA, Divers SG, Hoon DS, Kopetz ES (2015). Analytical and clinical validation of a digital sequencing panel for quantitative, highly accurate evaluation of cell-free circulating tumor DNA. PLoS One.

[57] Chan KC, Jiang P, Zheng YW, Liao GJ, Sun H, Wong J, Siu SS, Chan WC, Chan SL, Chan AT (2013). Cancer genome scanning in plasma: detection of tumor-associated copy number aberrations, single-nucleotide variants, and tumoral heterogeneity by massively parallel sequencing. Clin Chem.

[58] Liao W, Mao Y, Ge P, Yang H, Xu H, Lu X, Sang X, Zhong S (2015). Value of quantitative and qualitative analyses of circulating cell-free DNA as diagnostic tools for hepatocellular carcinoma: a meta-analysis. Medicine (Baltimore).

[59] Fujimoto A, Totoki Y, Abe T, Boroevich KA, Hosoda F, Nguyen HH, Aoki M, Hosono N, Kubo M, Miya F (2012). Whole-genome sequencing of liver cancers identifies etiological influences on mutation patterns and recurrent mutations in chromatin regulators. Nat Genet.

[60] Guichard C, Amaddeo G, Imbeaud S, Ladeiro Y, Pelletier L, Maad IB, Calderaro J, Bioulac-Sage P, Letexier M, Degos F (2012). Integrated analysis of somatic mutations and focal copy-number changes identifies key genes and pathways in hepatocellular carcinoma. Nat Genet.

[61] Ong CK, Subimerb C, Pairojkul C, Wongkham S, Cutcutache I, Yu W, McPherson JR, Allen GE, Ng CC, Wong BH (2012). Exome sequencing of liver fluke-associated cholangiocarcinoma. Nat Genet.

[62] Llovet JM, Villanueva A, Lachenmayer A, Finn RS (2015). Advances in targeted therapies for hepatocellular carcinoma in the genomic era. Nat Rev Clin Oncol.

[63] Schulze K, Imbeaud S, Letouze E, Alexandrov LB, Calderaro J, Rebouissou S, Couchy G, Meiller C, Shinde J, Soysouvanh F (2015). Exome sequencing of hepatocellular carcinomas identifies new mutational signatures and potential therapeutic targets. Nat Genet.

[64] Zucman-Rossi J (2010). Molecular classification of hepatocellular carcinoma. Dig Liver Dis.

[65] Yates LR, Campbell PJ (2012). Evolution of the cancer genome. Nat Rev Genet.

[66] Gerlinger M, Rowan AJ, Horswell S, Larkin J, Endesfelder D, Gronroos E, Martinez P, Matthews N, Stewart A, Tarpey P (2012). Intratumor heterogeneity and branched evolution revealed by multiregion sequencing. N Engl J Med.

[67] Patel KM, Tsui DW (2015). The translational potential of circulating tumour DNA in oncology. Clin Biochem.

[68] De Mattos-Arruda L, Weigelt B, Cortes J, Won HH, Ng CK, Nuciforo P, Bidard FC, Aura C, Saura C, Peg V (2014). Capturing intra-tumor genetic heterogeneity by de novo mutation profiling of circulating cell-free tumor DNA: a proof-of-principle. Ann Oncol.

[69] Tsuchiya N, Sawada Y, Endo I, Saito K, Uemura Y, Nakatsura T (2015). Biomarkers for the early diagnosis of hepatocellular carcinoma. World J Gastroenterol.

[70] Gormally E, Caboux E, Vineis P, Hainaut P (2007). Circulating free DNA in plasma or serum as biomarker of carcinogenesis: practical aspects and biological significance. Mutat Res.

[71] Jiang P, Chan CW, Chan KC, Cheng SH, Wong J, Wong VW, Wong GL, Chan SL, Mok TS, Chan HL (2015). Lengthening and shortening of plasma DNA in hepatocellular carcinoma patients. Proc Natl Acad Sci USA.

